# Serum bone metabolism biomarkers in predicting tumor bone metastasis risk and their association with cancer pain: a retrospective study

**DOI:** 10.3389/fpain.2025.1514459

**Published:** 2025-03-28

**Authors:** Sijia Zhang, Kai Huang, Tian Zhou, Yao Wang, Yunqing Xu, Quan Tang, Guangqin Xiao

**Affiliations:** ^1^ Cancer Center, Union Hospital, Tongji Medical College, Huazhong University of Science and Technology, Wuhan, China; ^2^ Institute of Radiation Oncology, Union Hospital, Tongji Medical College, Huazhong University of Science and Technology, Wuhan, China; ^3^ Hubei Key Laboratory of Precision Radiation Oncology, Wuhan, China; ^4^ Department of Biophysics, Center for Integrative Physiology and Molecular Medicine (CIPMM), School of Medicine, Saarland University, Homburg, Germany; ^5^ Department of Oncology, The Third People’s Hospital of Hubei Province, Jianghan University, Wuhan, China; ^6^ Department of Infectious Diseases, Union Hospital, Tongji Medical College, Huazhong University of Science and Technology, Wuhan, China; ^7^ Department of Gastroenterology, Wuhan No. 1 Hospital (Wuhan Hospital of Traditional and Western Medicine), Wuhan, China; ^8^ Department of Oncology, People’s Hospital of Huangpi District, Jianghan University, Wuhan, China; ^9^ Department of Oncology, Hubei Aerospace Hospital, Xiaogan, China; ^10^ Department of Epidemiology, Harvard T.H. Chan School of Public Health, Boston, MA, United States; ^11^ Clinical and Translational Epidemiology Unit, Massachusetts General Hospital and Harvard Medical School, Boston, MA, United States

**Keywords:** N-terminal mid fragment of osteocalcin (NMID), total N-terminal propeptide of type I procollagen (TPINP), C-terminal telopeptide of type I collagen β-special sequence (β-CTX), tumor bone metastasis, numeric rating scale (NRS)

## Abstract

**Background:**

This study aims to develop a novel nomogram predictive model utilizing serum bone metabolism biomarkers to accurately predict and diagnose tumor bone metastasis. The creation of this model holds significant clinical implications, supporting the development of targeted intervention strategies, providing robust laboratory data, and guiding early patient treatment.

**Methods:**

A retrospective cohort study was conducted involving 266 patients treated at hospitals from September 2021 to January 2024. Patients were classified into three groups based on disease characteristics: tumor patients without bone metastasis, tumor patients with bone metastasis, and a control group consisting of individuals with neither tumor nor bone metabolism-related conditions. The primary serum bone metabolism biomarkers assessed included the N-terminal mid fragment of osteocalcin (NMID), the total N-terminal propeptide of type I procollagen (TPINP), and the C-terminal telopeptide of type I collagen β-special sequence (β-CTX). Multivariate statistical methods, including logistic regression and Cox regression, were employed for data analysis, while the nomogram model was rigorously evaluated using a variety of tools such as receiver operating characteristic (ROC) curves.

**Results:**

The study found that the levels of NMID, TPINP, and β-CTX were significantly elevated in patients with bone metastasis compared to the other groups. These biomarkers were strongly associated with the incidence of tumor bone metastasis and identified as independent risk factors for this condition. The nomogram model demonstrated exceptional predictive performance, characterized by high area under the AUC values, robust time-dependent ROC curves, accurate calibration curves, and effective decision curve analysis. Notably, a positive correlation was observed between NMID, TPINP, β-CTX, and numeric rating scale (NRS) pain scores, providing valuable biomarkers for evaluating and managing pain associated with tumor bone metastasis.

**Conclusion:**

This study successfully established a nomogram predictive model based on serum bone metabolism biomarkers, with NMID, TPINP, and β-CTX emerging as critical indicators. The correlation between these biomarkers and NRS pain scores offers a novel understanding of the pain mechanisms associated with tumor bone metastasis, providing clinicians with essential reference points for diagnostic and therapeutic decision-making, thereby enhancing the practical application of the model in clinical settings.

## 1 Introduction

With the relentless advancements in medical technology, there has been a significant increase in the survival period of patients with malignant tumors (1–3). However, this improvement is coupled with an increased incidence of tumor bone metastasis. Bones, ranking as one of the top three common sites for malignant tumor metastasis after the lungs and liver, show a metastasis rate that is 35–40 times higher than that of primary bone tumors (4–6). This prevalence is particularly notable in cancers such as breast, lung, prostate, gastric, and thyroid cancers, with the highest occurrences observed in lung, breast, and prostate cancers (4–6). The diagnosis of tumor bone metastasis predominantly relies on bone tissue biopsy or various imaging techniques (7, 8). However, these methods, often hampered by their invasive nature or lack of sufficient sensitivity, frequently fall short in achieving the early diagnosis and timely intervention of tumor bone metastasis. In this context, bone metabolism biomarkers have emerged as vital indicators. These biomarkers, reflecting the rate of bone absorption and formation, provide insights into the extent of bone destruction and repair. During tumor bone metastasis, cytokines disrupt the normal bone metabolism by acting on osteoblastic and osteoclastic cells, leading to abnormal fluctuations in bone metabolism biomarkers (9, 10). One common complication among tumor patients is cancer pain, specifically pain associated with bone metastasis. Epidemiological data indicate that approximately 80% of patients with malignacnt tumors suffer from symptoms of bone metastasis pain (11–13). This pain is intricately linked to factors such as tumor invasion into the bone, compression of nerve tissues, and the destruction of bone caused by metastasis (11). Bone metastasis pain, a prevalent and often intolerable symptom in patients with malignant tumors, tends to intensify post-radiotherapy and chemotherapy (14, 15). Chronic bone metastasis pain can evolve into intractable pain, exacerbating symptoms like fatigue, appetite loss, anxiety, and depression, thus profoundly affecting the patients' quality of life (16, 17). In the mechanism of malignant tumor bone metastasis, the spread of cancer cells to bone tissues alters the bone's microenvironment, disrupting the equilibrium among bone cells and leading to significant pain symptoms (18). Bone metabolism biomarkers, which are released into the bloodstream during bone metabolism, become imbalanced in cases of bone metastasis (19, 20). Among the current diagnostic methods for tumor bone metastasis, x-rays, MR scans, and CT scans are extensively utilized, yet they have their limitations. Bone metabolic biomarkers are increasingly recognized for their convenience and sensitivity. Serum biomarkers like N-terminal osteocalcin (NMID), total type I collagen N-terminal propeptide (TPINP), and Beta-C-terminal telopeptide (β-CTX) serve as crucial indicators of osteoblast activity, bone formation and resorption rates, and bone matrix absorption and osteoclast activity respectively (21–24).

This study is dedicated to exploring the relationship between serum bone metabolism biomarkers (NMID, TPINP, and β-CTX) in predicting tumor bone metastasis and their correlation with cancer pain. By conducting an in-depth investigation of these biomarkers, we aim to develop new strategies for early diagnosis and intervention in tumor bone metastasis. Additionally, we seek to establish more effective methods for assessing and treating cancer pain. Our research endeavors to enhance the understanding of the mechanisms underlying tumor bone metastasis and the associated pain, thereby facilitating the formulation of more comprehensive and precise treatment plans for patients with malignant tumors. Ultimately, our objective is to improve the quality of life and treatment outcomes for these patients, addressing not only the physical aspects of their condition but also the overall impact on their well-being. Through this study, we hope to contribute significantly to the field of oncology, particularly in the management of bone metastases and the alleviation of cancer-related pain.

## 2 Material and methods

### 2.1 Study design and participants

This retrospective cohort study was conducted at Union Hospital, Tongji Medical College, Huazhong University of Science and Technology, and The Third People's Hospital of Hubei Province, encompassing patients treated from September 2021 to January 2024. A total of 266 patients were enrolled, categorized into three groups: the non-bone metastasis tumor group (*n* = 90), the bone metastasis tumor group (*n* = 87), and a control group consisting of individuals with non-tumor, non-bone metabolism-related benign diseases (*n* = 89). Tumor diagnoses were confirmed through a combination of histopathological examination, medical imaging, and cytological testing. Inclusion criteria were as follows: (1) patients histologically diagnosed with malignant tumors based on surgical pathology; (2) whole-body bone scans using ECT, with suspicious areas further evaluated using x-ray, CT, or MRI scans, and bone metastasis confirmed by at least two senior physicians. Exclusion criteria included: (1) the presence of bone metabolic diseases; (2) use of bisphosphonates, corticosteroids, or calcium supplements within four weeks prior to screening; (3) traumatic bone fractures occurring within the past 90 days; (4) receipt of radiotherapy in the four weeks preceding screening. The study was approved by the Ethics Committees of Union Hospital, Tongji Medical College, Huazhong University of Science and Technology, and The Third People's Hospital of Hubei Province (Ethics Approval Number: LW2023008).

### 2.2 Comprehensive data collection

The clinical pathological features of the patients, including basic disease, gender, smoking, drinking, histology, T stage, N stage, M stage, clinical stage, bone metastasis, age, NRS pain scores, pain medication, dose of pain medication, frequency of pain medication, survival status, overall survival (OS) were collected from medical records. Blood indices including neutrophils (Neu), lymphocytes (Lym), monocytes (Mon), platelets (PLT), C-reactive protein (CRP), lactate dehydrogenase (LDH), uric acid (UA), albumin (ALB), alanine transaminase (ALT), aspartate transaminase (AST), alkaline phosphatase (ALP), calcium (Ca^2+^), NMID, TPINP, β-CTX, inflammatory burden index (IBI), systemic immune-inflammation index (SII), prognostic nutritional index (PNI), neutrophil to lymphocyte ratio (NLR), platelet to lymphocyte ratio (PLR), lymphocyte to monocyte ratio (LMR), C-reactive protein to albumin ratio (CAR), aspartate platelet ratio index (APRI), AST/ALT ratio (AAR), and inflammation-immunity-nutrition score (IINS) were measured within one week after diagnosis through routine blood tests. Pathological staging was confirmed according to the 8th edition of the American Joint Committee on Cancer (AJCC). No patients underwent emergency resection. Treatment for the patients was conducted in accordance with the guidelines of the National Health Commission of the People's Republic of China.

### 2.3 Rigorous follow-up protocol

After diagnosis, we obtained outcomes by reviewing medical records and making follow-up calls every 3–6 months for the first and second years, and then every 6 months for the third to fifth years. The primary outcome was OS. OS was defined as the interval from the date of tumor diagnosis to the date of death, lost to follow-up, or the end of the follow-up (January 2024), whichever came first.

### 2.4 Optimal cut-off determination

We selected the optimum cut-offs for NMID, TPINP, β-CTX, AAR, APRI, CAR, IBI, LMR, NLR, PLR, PNI, SII and INNS using X-tile software version 3.6.1 (https://medicine.yale.edu/lab/rimm/research/software/, Yale University School of Medicine, New Haven, CT) based on the association between each indicator with the patients' OS.

### 2.5 Statistical analysis

Continuous variables were presented as median or mean. The student's *t*-test or the Wilcoxon rank sum test was used for comparisons between groups with continuous variables. Categorical variables were expressed by counts and percentages. The chi-square test was used for comparison between groups with categorical variables, and the Fisher's exact test was used when the counts were limited. The area under the curves (AUCs) and Harrell's concordance indices (C-indices) of the indicators were estimated and compared by *q* values (adjusted *p* values by Benjamini & Hochberg method). The AUCs and C-indices were calculated based on logistic regression models and Cox regression models, respectively. Therefore, to demonstrate the prognostic performances of indicators well, the AUCs and C-indices were calculated together. Time-dependent ROC curves, time-AUC curves, decision curve analysis (DCA), Kaplan–Meier survival curves and Log rank tests were used to detect the prognostic performance. Compared with ordinary ROC curve, time-dependent ROC curve could observe the prognostic performance of indicator at a specific point in time after operation. Furthermore, time-AUC curve could observe the dynamic prognostic performance of indicator at all point in time after operation. Decision curve analysis is conducted to determine the clinical usefulness of the indicator via quantifying the net benefits at different threshold probabilities. Univariate and multivariate Cox proportional hazards regression were applied to detect the associations of individual clinicopathological indicators with OS by calculating hazard ratios (HRs) and 95% confidence intervals (CIs). All statistical tests were two-sided, and *p* < 0.05 was considered statistically significant. Time-dependent ROC curves, time-AUC curves, decision curves, Kaplan–Meier survival curves, C-indices, and forest plots were performed using packages “survivalROC”, “timeROC”, “ggDCA”, “survminer”, “survival”, and “forestplot” of R 3.6.0 (The R Foundation for Statistical Computing, Vienna, Austria), respectively. Other statistical analyses were performed using SAS Statistics software 9.4 (SAS Institute Inc, Cary, North Carolina, USA).

## 3 Results

### 3.1 Baseline characteristics of patients

Between September 2021 and January 2024, 266 patients were enrolled in the study. These individuals were divided into three groups: 90 patients with tumors without bone metastasis, 87 patients with tumors with bone metastasis, and 89 patients in a control group suffering from benign diseases unrelated to tumors or bone metabolism. The gender distribution among these groups included 93 males (35%) and 173 females (65%). 124 patients (46.6%) were under 65 years, and 142 patients (53.4%) were 65 years or older. A substantial portion of the cohort, 170 patients (63.9%), had a history of comorbidities, whereas 96 patients (36.1%) had no such history. Smoking habits were reported with 35 patients (13.2%) being smokers and 231 patients (86.8%) non-smokers. In terms of biochemical markers, NMID levels were <16.5 ng/ml in 157 patients (59%) and ≥16.5 ng/ml in 109 patients (41%). TPNIP levels were <58 ng/ml in 147 patients (55.3%) and ≥58 ng/ml in 119 patients (44.7%). β-CTX levels were <515 pg/ml in 167 patients (62.8%) and ≥515 pg/ml in 99 patients (37.2%). Within the groups with and without bone metastasis, there were 54 cases (30.5%) of lung cancer, 37 cases (20.9%) of gastrointestinal tumors, 19 cases (10.7%) of reproductive system tumors, 6 cases (3.4%) of endocrine system tumors, 16 cases (9%) of hematological malignancies, and 45 cases (25.4%) of other types. The histopathological types were 144 cases (81.4%) of adenocarcinoma, 9 cases (5.1%) of squamous cell carcinoma, and 24 cases (13.6%) of small cell carcinoma. In terms of clinical staging, 13 patients (7.3%) were in stage III, and 164 patients (92.7%) were in stage IV. Regarding pain management, 90 patients (50.8%) did not use analgesics, 53 patients (29.9%) used non-steroidal anti-inflammatory drugs (NSAIDs), 17 patients (9.6%) used weak opioids, and 17 patients (9.6%) used strong opioids. The daily dosage of pain medication varied: 32 patients (18.1%) received 10–15 mg, 98 patients (55.4%) received 30–40 mg, 15 patients (8.5%) received 50 mg, and 32 patients (18.1%) received 100–120 mg. The frequency of analgesic administration was qd (once daily) in 7 cases (4%), q6 h (every 6 h) in 4 cases (2.3%), q8 h (every 8 h) in 10 cases (5.6%), and q12 h (every 12 h) in 156 cases (88.1%) (Supplementary Table S1).

### 3.2 Analysis of bone metabolism markers in patients with malignant tumors

**Figure 1:**
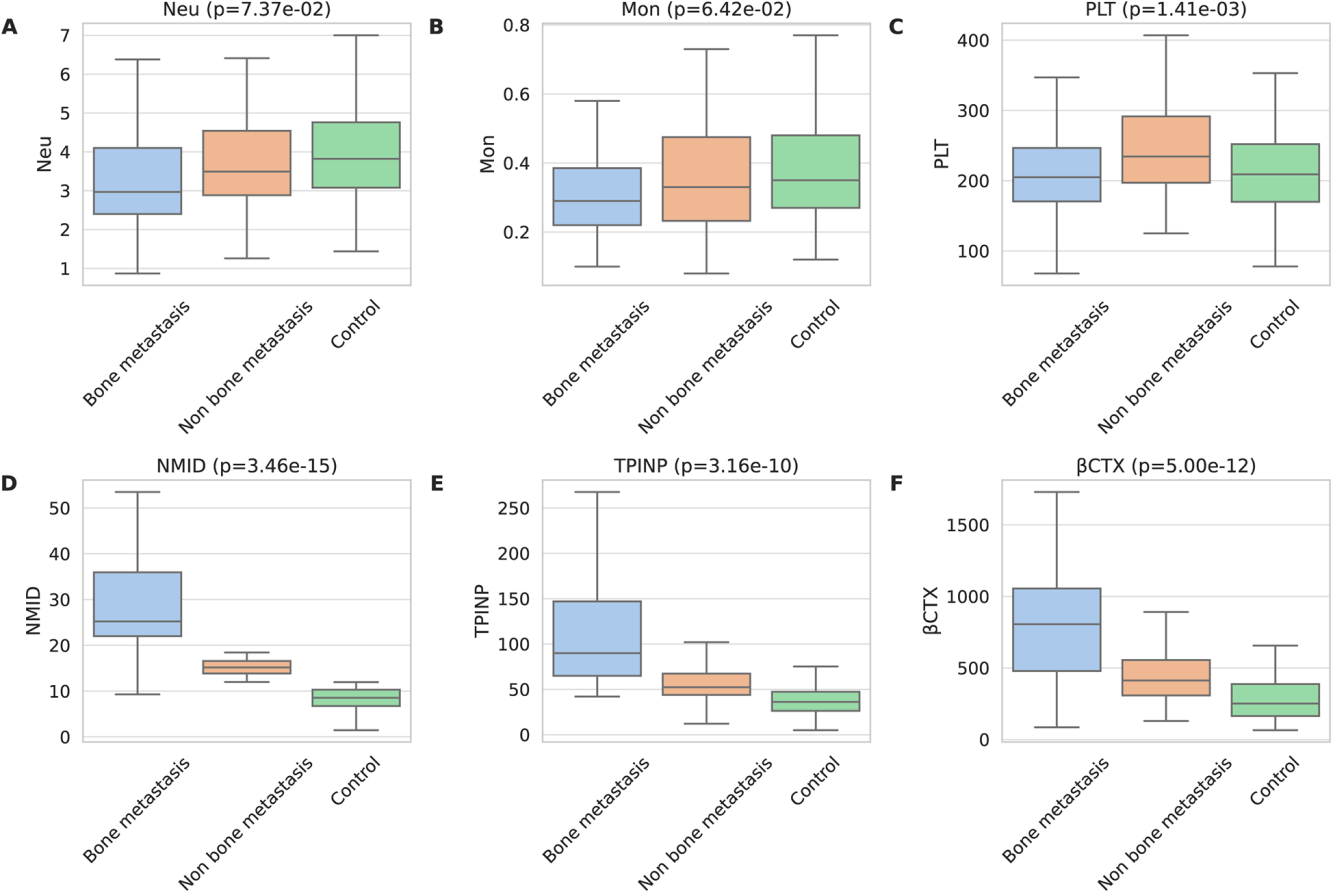
Comprehensive analysis of bone metabolism markers in patients with malignant tumors. **(A)** Neu. **(B)** Mon. **(C)** PLT. **(D)** NMID. **(E)** TPINP. **(F)** β-CTX.

In the tumor groups, both with and without bone metastasis, Neu and Mon counts were significantly lower than in the control group (*p* = 7.37 × 10^−2^ and *p* = 6.42 × 10^−2^) (Figures 1A,B). Notably, the PLT count in the group without bone metastasis was higher than that in both the bone metastasis group and the control group (*p* = 1.41 × 10^−03^) (Figure 1C). Additionally, when comparing bone metabolism markers among the groups, those with tumor bone metastasis displayed significantly higher levels of NMID (*p* = 3.46 × 10^−15^), TPINP (*p* = 3.16 × 10^−10^), and β-CTX (*p* = 5.00 × 10^−12^) than both the group without bone metastasis and the control group (Figures 1D–F). The remaining laboratory indices did not show any statistically significant differences.

### 3.3 Association of clinicopathological features and laboratory parameters with tumor bone metastasis, NMID, TPINP, and β-CTX levels

**Figure 2:**
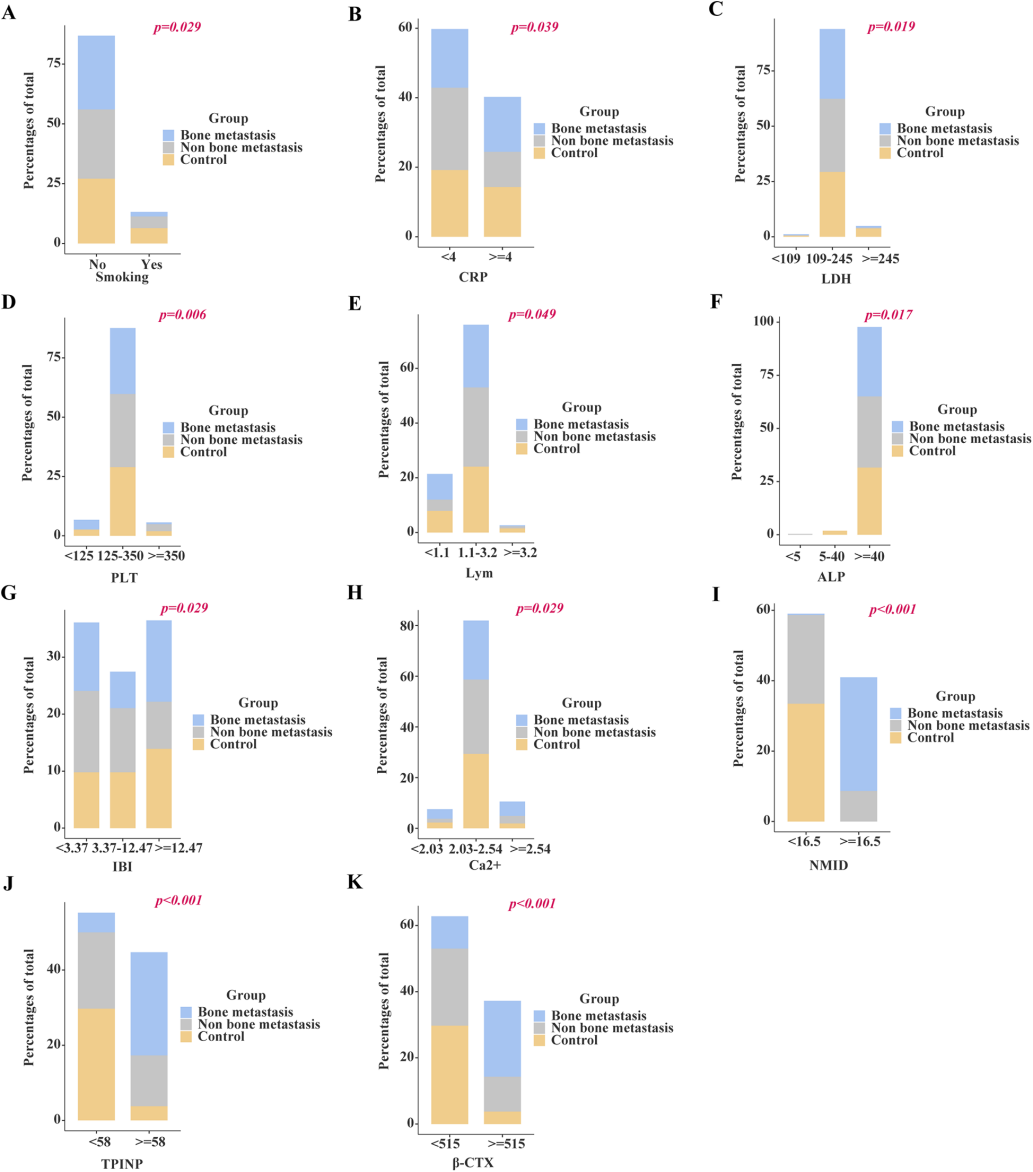
Chi-square analysis revealed significant associations between several variables and the occurrence of bone metastasis in tumors. **(A)** Smoking history. **(B)** CRP levels. **(C)** LDH levels. **(D)** PLT count. **(E)** Lym count. **(F)** ALP levels. **(G)** IBI levels. **(H)** Ca^2+^. **(I)** NMID. **(J)** TPINP. **(K)** β-CTX.

**Figure 3:**
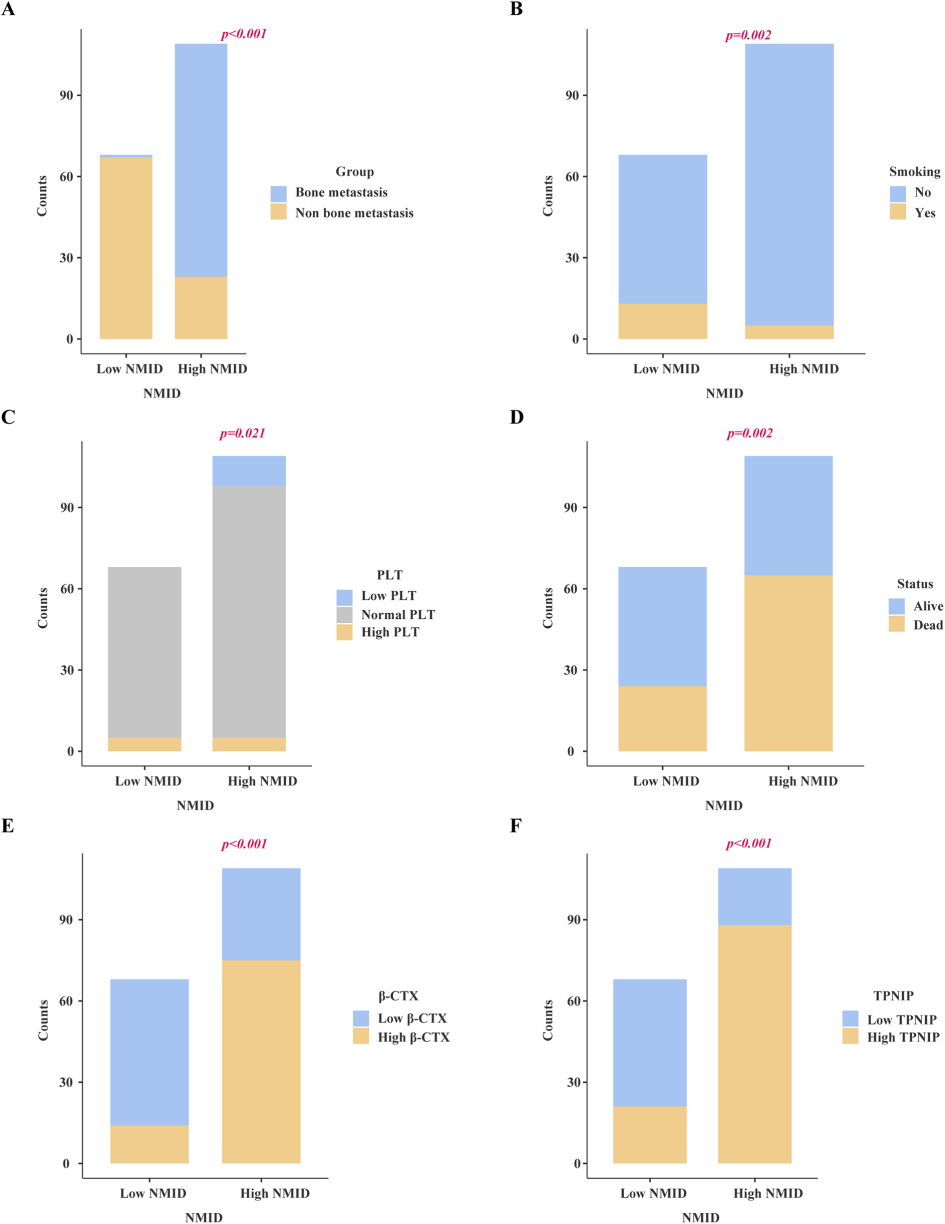
Chi-square analysis revealed significant associations between several variables and the levels of NMID. **(A)** Bone metastasis. **(B)** Smoking history. **(C)** PLT count. **(D)** Status. **(E)** β-CTX. **(F)** TPINP.

**Figure 4:**
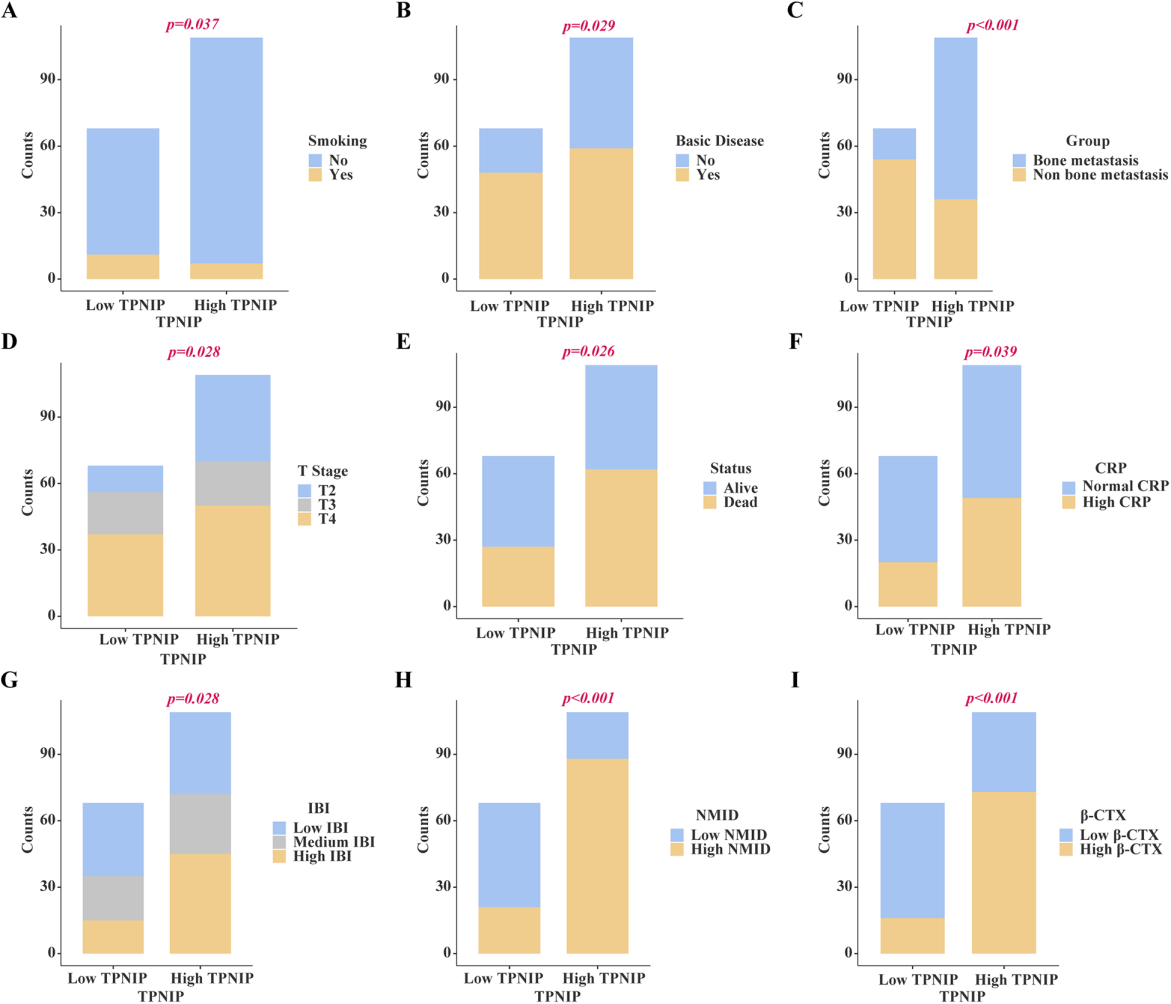
Chi-square analysis revealed significant associations between several variables and the levels of TPINP. **(A)** Smoking history. **(B)** Basic disease. **(C)** Bone metastasis. **(D)** T stage. **(E)** Status, **(F)** CRP. **(G)** IBI. **(H)** NMID. **(I)** β-CTX.

**Figure 5:**
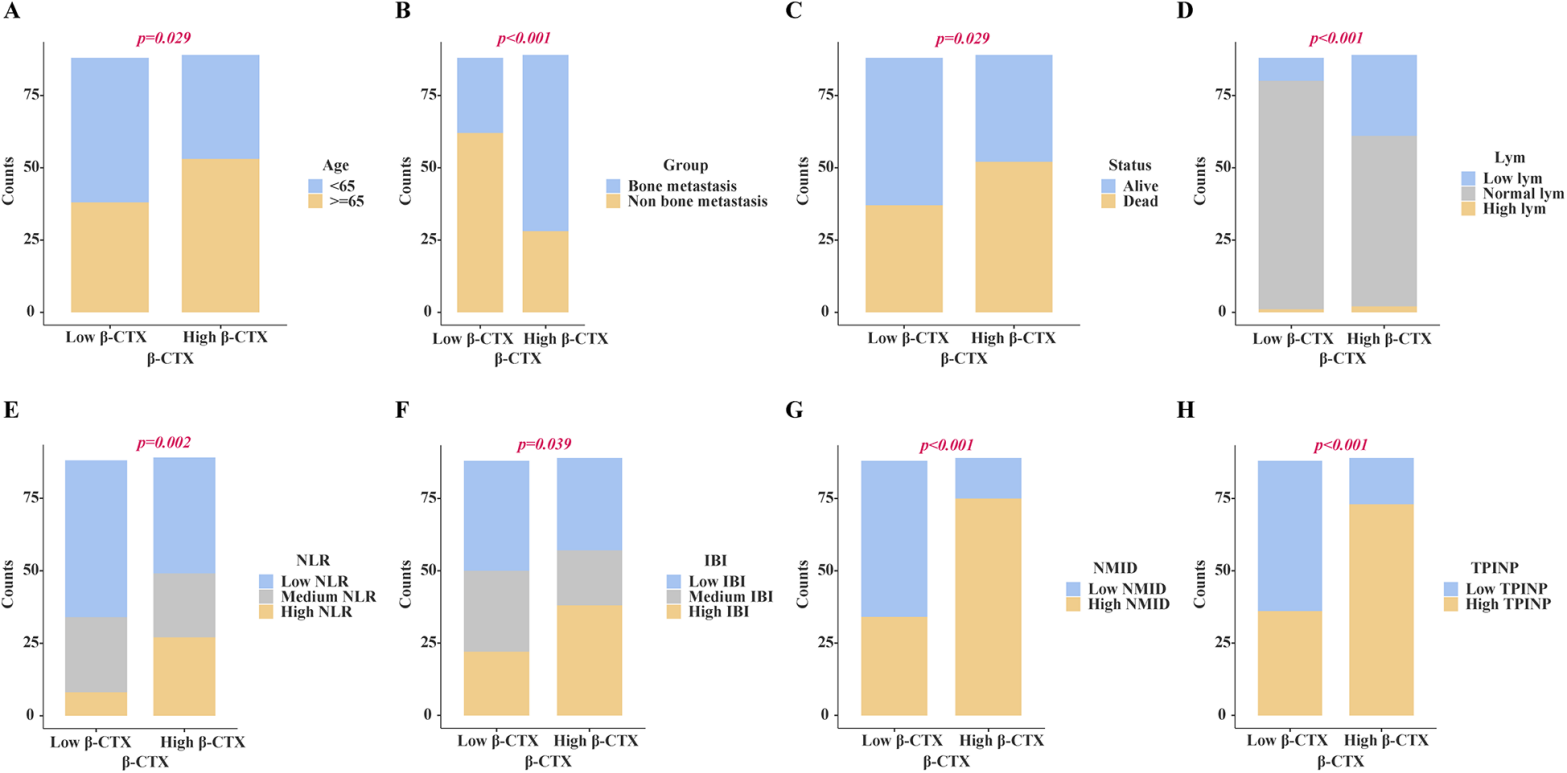
Chi-square analysis revealed significant associations between several variables and the levels of β-CTX. **(A)** Age. **(B)** Bone metastasis. **(C)** Status. **(D)** Lym count. **(E)** NLR. **(F)** IBI. **(G)** NMID. **(H)** TPINP.

Chi-square analysis revealed significant associations between several variables and the occurrence of bone metastasis in tumors. These variables include pre-existing medical conditions, smoking history, CRP levels, LDH levels, PLT count, Lym count, ALP levels, IBI levels, Ca^2+^, NMID, TPINP, and β-CTX (Figures 2A–K, Supplementary Table S2). Elevated levels of NMID were significantly correlated with the incidence of bone metastasis, smoking history, PLT count, TPINP, β-CTX, and status (Figures 3A–F, Supplementary Table S3). Variations in TPINP levels showed significant associations with the presence of bone metastasis, basic disease, smoking history, T staging, CRP levels, NMID, β-CTX, IBI, and status (Figures 4A–I, Supplementary Table S4). Furthermore, the expression of β-CTX was significantly linked to bone metastasis, age, Lym count, NMID, TPINP, IBI, NLR, and status (Figures 5A–H, Supplementary Table S5).

### 3.4 Survival analysis

**Figure 6:**
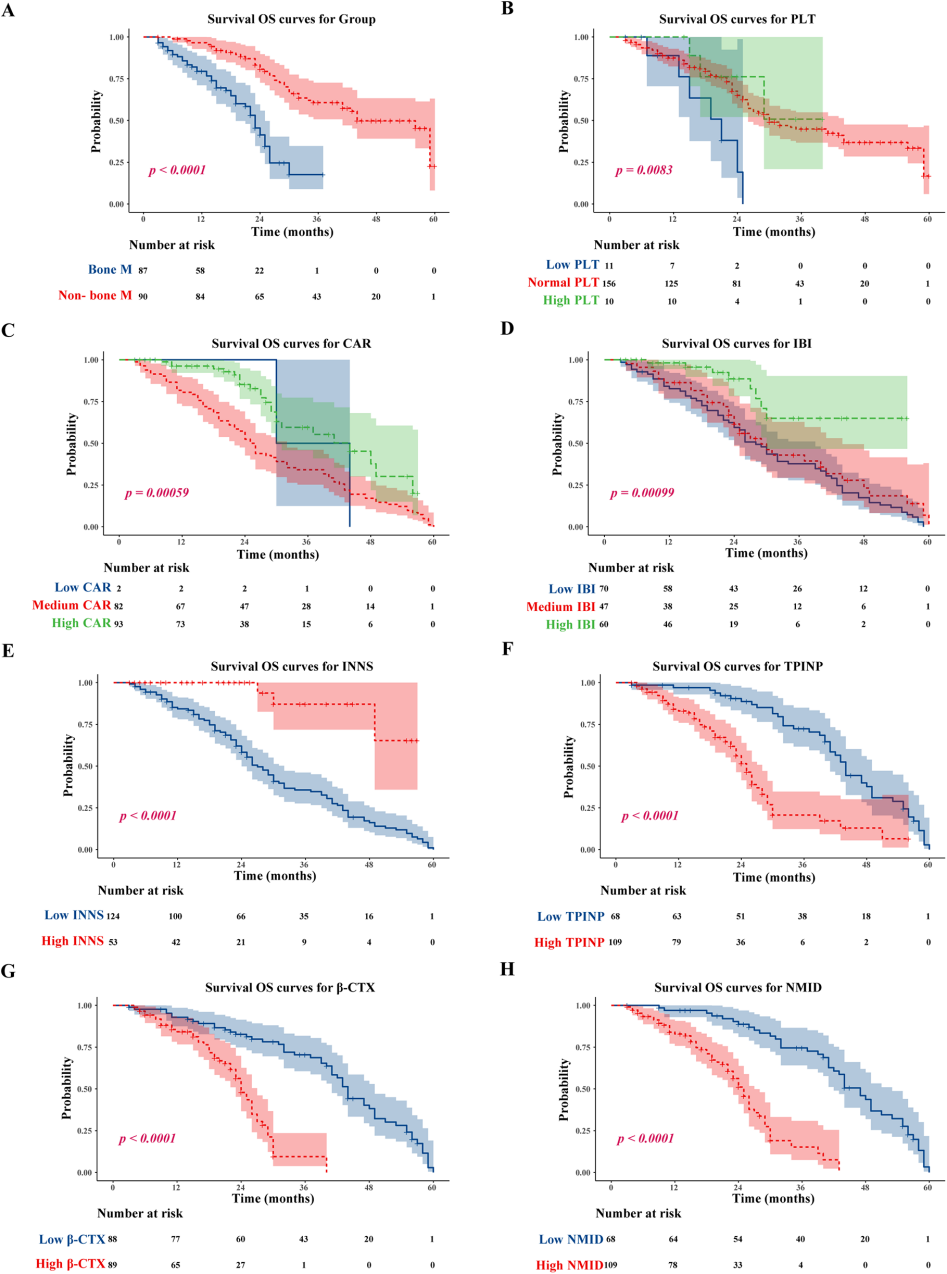
Tumor bone metastasis, low PLT, low CAR; low IBI, low INNS, and high TPINP, β-CTX, NMID were associated with worse OS. **(A)** OS for tumor bone metastasis or non-tumor bone metastasis patients. **(B)** OS for PLT. **(C)** OS for CAR. **(D)** OS for IBI. **(E)** OS for INNS. **(F)** OS for levels of TPINP. **(G)** OS for levels of β-CTX. **(H)** OS for levels of NMID.

A significant difference was observed in OS between tumor patients with and without bone metastasis, with the former group demonstrating markedly shorter survival times (*p* < 0.0001) (Figure 6A). Notably, patients exhibiting high NMID expression within their tumors had significantly reduced OS compared to those with low NMID expression (Figure 6H). Similarly, elevated TPINP expression in tumor patients correlated with shorter OS, in contrast to those with lower TPINP levels (Figure 6F). Additionally, increased β-CTX expression in tumors was associated with a significant decrease in OS compared to patients with lower β-CTX levels (Figure 6G). In the context of the CAR, higher expression levels were linked to shorter OS compared to lower CAR levels (Figure 6C). In a contrasting trend, higher IBI expression was indicative of longer OS, as opposed to lower IBI levels (Figure 6D). Furthermore, patients with higher INNS expression in their tumors experienced significantly prolonged OS compared to those with lower INNS expression (Figure 6E). Finally, patients with lower PLT count in tumors showed significantly reduced OS compared to those with higher PLT levels (Figure 6B).

### 3.5 Selection of relevant independent prognostic factors

**Figure 7:**
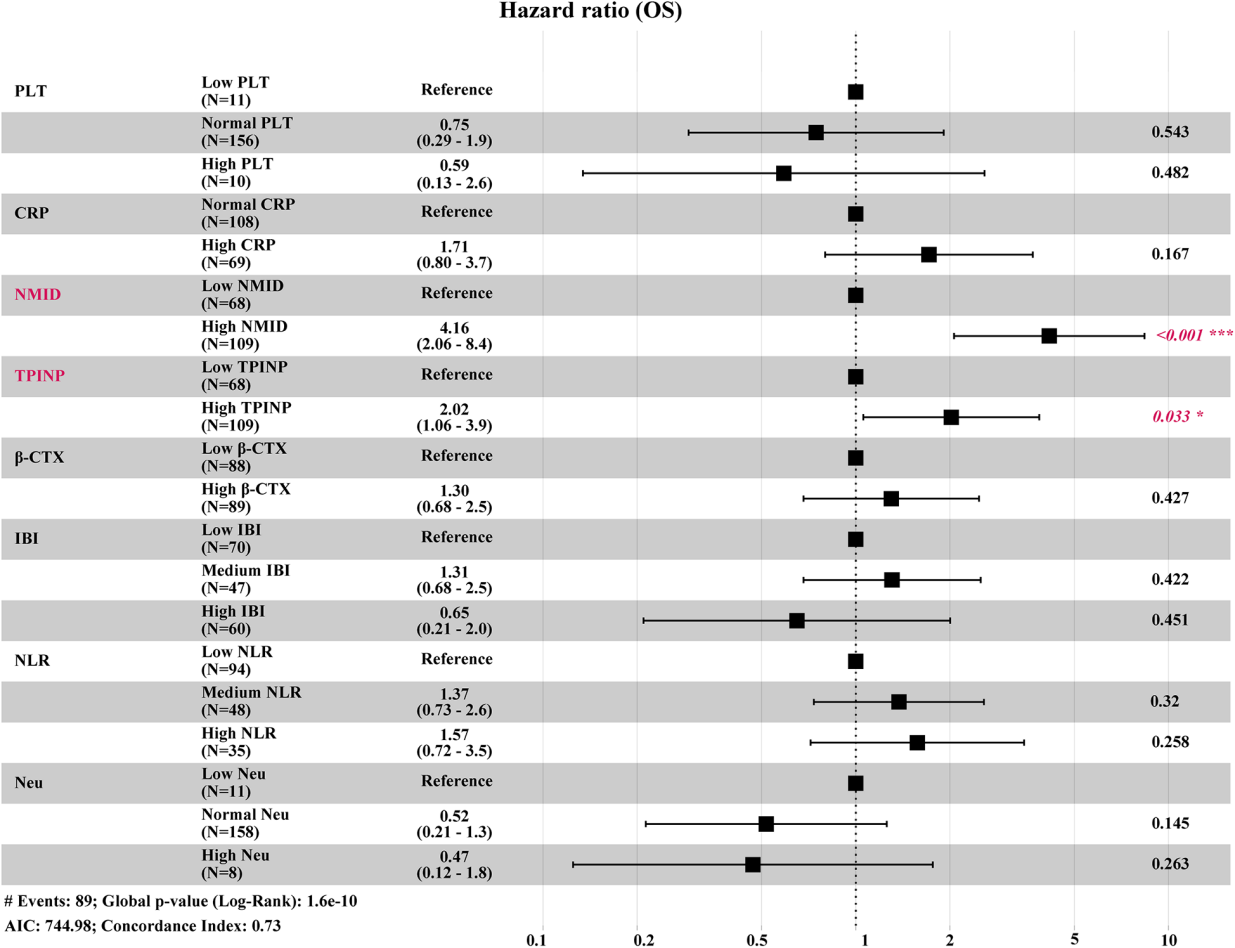
Multivariate analysis identified NMID and TPINP as independent risk factors for OS in cancer patients, as illustrated in the forest plot for OS.

In this study, the median follow-up duration was 23.0 months. By the end of this period, 89 patients (50.3%) had passed away. Our findings revealed that in univariate Cox regression analyses, several parameters including Neu, PLT, CRP, NMID, TPINP, β-CTX, IBI, and NLR showed statistically significant differences (Table 1). Subsequent multivariate analysis further identified NMID and TPINP as independent risk factors for OS in cancer patients (Figure 7, Table 2). This underscores their pivotal role in predictive modeling, emphasizing the importance of these biomarkers not only as diagnostic tools but also as critical prognostic indicators in cancer management. Their relevance in the clinical setting offers valuable insights into the disease progression and patient outcomes, and these findings could potentially guide therapeutic decisions and patient counseling in oncological practice.

**Table 1:** Clinicopathologic characteristics and single-factor cox regression analysis for OS in patients with and without bone metastasis in tumors.

Features		All	HR (Univariable)
Basic disease	No	70	–
	Yes	107	0.74 (0.49–1.13, *p* = 0.165)
Gender	Male	58	–
	Female	119	0.84 (0.55–1.30, *p* = 0.442)
Smoking	No	159	–
	Yes	18	0.47 (0.21–1.09, *p* = 0.079)
Drinking	No	166	–
	Yes	11	0.45 (0.14–1.43, *p* = 0.175)
Diagnostic	Lung cancer	54	–
	Gastrointestinal tumor	37	0.67 (0.35–1.28, *p* = 0.222)
	Genitourinary tumor	19	0.72 (0.33–1.59, *p* = 0.417)
	Endocrine system tumor	6	1.59 (0.55–4.56, *p* = 0.389)
	Hematological system tumor	16	1.73 (0.81–3.69, *p* = 0.156)
	Other	45	1.19 (0.70–2.04, *p* = 0.524)
Pathology	Adenocarcinoma	144	–
	Squamous Cell Carcinoma	9	0.50 (0.16–1.60, *p* = 0.245)
	Small Cell	24	0.85 (0.45–1.61, *p* = 0.626)
T stage	T2	51	–
	T3	39	0.68 (0.38–1.21, *p* = 0.192)
	T4	87	0.62 (0.38–1.00, *p* = 0.048)
N stage	N0	11	–
	N1	14	1.70 (0.58–4.92, *p* = 0.331)
	N2	68	1.51 (0.64–3.59, *p* = 0.350)
	N3	84	1.07 (0.45–2.54, *p* = 0.881)
M stage	M0	21	–
	M1	156	1.29 (0.67–2.49, *p* = 0.454)
Clinical stage	3	13	–
	4	164	1.21 (0.56–2.63, *p* = 0.630)
Bone metastasis	Single	75	–
	Multiple	102	1.29 (0.84–1.99, *p* = 0.238)
Age	<65	86	–
	≥65	91	1.05 (0.69–1.59, *p* = 0.834)
Neu	Low neu	11	–
	Normal neu	158	0.47 (0.23–0.98, *p* = 0.044)
	High neu	8	0.62 (0.18–2.05, *p* = 0.429)
Lym	Low lym	36	–
	Normal lym	138	0.66 (0.40–1.10, *p* = 0.113)
	High lym	3	0.84 (0.19–3.61, *p* = 0.811)
Mon	Low mon	1	–
	Normal mon	165	0.28 (0.04–2.03, *p* = 0.208)
	High mon	11	0.28 (0.03–2.34, *p* = 0.238)
PLT	Low PLT	11	–
	Normal PLT	156	0.31 (0.14–0.69, *p* = 0.004)
	High PLT	10	0.23 (0.06–0.89, *p* = 0.034)
CRP	Normal CRP	108	–
	High CRP	69	1.62 (1.05–2.50, *p* = 0.029)
LDH	Low LDH	2	–
	Normal LDH	172	92,11,736.79 (0.00-Inf, *p* = 0.996)
	High LDH	3	45,59,595.28 (0.00-Inf, *p* = 0.996)
UA	Normal UA	1	–
	High UA	176	33,15,932.51 (0.00-Inf, *p* = 0.995)
ALB	Low ALB	21	–
	Normal ALB	154	1.52 (0.70–3.28, *p* = 0.292)
	High ALB	2	1.64 (0.20–13.33, *p* = 0.645)
ALT	Normal ALT	167	–
	High ALT	10	1.19 (0.48–2.94, *p* = 0.705)
AST	Low AST	3	–
	Normal AST	164	92,99,575.51 (0.00-Inf, *p* = 0.994)
	High AST	10	82,82,066.37 (0.00-Inf, *p* = 0.994)
ALP	Low ALP	1	–
	High ALP	176	0.43 (0.06–3.12, *p* = 0.405)
Ca^2+^	Low Ca^2+^	14	–
	Normal Ca^2+^	140	0.77 (0.38–1.54, *p* = 0.455)
	High Ca^2+^	23	1.11 (0.47–2.60, *p* = 0.809)
NMID	Low NMID	68	–
	High NMID	109	6.34 (3.61–11.14, *p* < 0.001)
TPINP	Low TPINP	68	–
	High TPINP	109	4.17 (2.53–6.88, *p* < 0.001)
β-CTX	Low β-CTX	88	–
	High β-CTX	89	4.00 (2.44–6.56, *p* < 0.001)
IBI	Low IBI	70	–
	Medium IBI	47	1.46 (0.87–2.43, *p* = 0.152)
	High IBI	60	1.85 (1.11–3.07, *p* = 0.018)
SII	Low SII	20	–
	Medium SII	39	0.90 (0.46–1.78, *p* = 0.767)
	High SII	118	0.64 (0.35–1.18, *p* = 0.150)
PNI	Low PNI	19	–
	Medium PNI	133	1.47 (0.64–3.40, *p* = 0.365)
	High PNI	25	2.08 (0.82–5.30, *p* = 0.123)
NLR	Low NLR	94	–
	Medium NLR	48	1.20 (0.73–1.98, *p* = 0.470)
	High NLR	35	1.70 (1.01–2.88, *p* = 0.047)
PLR	Low PLR	20	–
	Medium PLR	95	0.78 (0.42–1.43, *p* = 0.422)
	High PLR	62	0.55 (0.28–1.07, *p* = 0.077)
LMR	Low LMR	44	–
	Medium LMR	109	0.99 (0.58–1.68, *p* = 0.962)
	High LMR	24	1.81 (0.95–3.44, *p* = 0.069)
CAR	Low CAR	2	–
	Medium CAR	82	75,61,416.28 (0.00-Inf, *p* = 0.994)
	High CAR	93	11,759,836.81 (0.00-Inf, *p* = 0.994)
APRI	Low APRI	38	–
	Medium APRI	61	0.88 (0.46–1.68, *p* = 0.701)
	High APRI	78	1.71 (0.96–3.04, *p* = 0.070)
AAR	Low AAR	49	–
	Medium AAR	44	1.08 (0.62–1.90, *p* = 0.777)
	High AAR	84	0.80 (0.49–1.31, *p* = 0.371)
INNS	Low IINS	124	–
	High INNS	53	1.40 (0.89–2.19, *p* = 0.146)
Painkillers	No use	90	–
	NSAIDs	53	0.99 (0.60–1.65, *p* = 0.983)
	Weak Opioids	17	1.46 (0.73–2.89, *p* = 0.281)
	Strong Opioids	17	0.84 (0.41–1.74, *p* = 0.642)
Painkiller dosage	10–15 mg	32	–
	30–40 mg	98	1.12 (0.62–2.04, *p* = 0.707)
	50 mg	15	1.21 (0.50–2.91, *p* = 0.673)
	100–120 mg	32	1.39 (0.68–2.85, *p* = 0.366)
Painkiller administration schedule	Qd	7	–
	q6 h	4	1.49 (0.21–10.72, *p* = 0.690)
	q8 h	10	2.85 (0.59–13.76, *p* = 0.193)
	q12 h	156	2.46 (0.60–10.04, *p* = 0.210)

Neu, neutrophils; Lym, lymphocytes; Mon, monocytes; PLT, platelets; CRP, C-reactive protein; LDH, lactate dehydrogenase; UA, uric acid; ALB, albumin; ALT, alanine aminotransferase; AST, aspartate aminotransferase; ALP, alkaline phosphatase; Ca^2+^, calcium; NMID, N-terminal mid, fragment of osteocalcin; TPINP, total procollagen type 1 N-terminal propeptide; β-CTX, β-C-terminal telopeptide of type 1 collagen; IBI, inflammatory burden index; SII, Systemic immune inflammation index; PNI, prognostic nutritional index; NLR, neutrophil to lymphocyte ratio; PLR, platelet to lymphocyte ratio; LMR, lymphocyte to monocyte ratio; CAR, CRP to albumin ratio; APRI, AST to platelet ratio index; AAR, AST to ALT ratio; INNS, inflammation immunity nutrition score.

**Table 2:** Multivariate cox regression analysis of clinically significant factors for OS in patients with and without bone metastasis in tumors.

Features		All	HR (univariable)	HR (multivariable)
Neu	Low neu	11	–	–
	Normal neu	158	0.47 (0.23–0.98, *p* = 0.044)	0.52 (0.21–1.26, *p* = 0.145)
	High neu	8	0.62 (0.18–2.05, *p* = 0.429)	0.47 (0.12–1.76, *p* = 0.263)
PLT	Low PLT	11	–	–
	Normal PLT	156	0.31 (0.14–0.69, *p* = 0.004)	0.75 (0.29–1.91, *p* = 0.543)
	High PLT	10	0.23 (0.06–0.89, *p* = 0.034)	0.59 (0.13–2.58, *p* = 0.482)
CRP	Normal CRP	108	–	–
	High CRP	69	1.62 (1.05–2.50, *p* = 0.029)	1.71 (0.80–3.68, *p* = 0.167)
NMID	Low NMID	68	–	–
	High NMID	109	6.34 (3.61–11.14, *p* < 0.001)	4.16 (2.06–8.38, *p* < 0.001)
TPINP	Low TPINP	68	–	–
	High TPINP	109	4.17 (2.53–6.88, *p* < 0.001)	2.02 (1.06–3.86, *p* = 0.033)
β-CTX	Low β-CTX	88	–	–
	High β-CTX	89	4.00 (2.44–6.56, *p* < 0.001)	1.30 (0.68–2.48, *p* = 0.427)
IBI	Low IBI	70	–	–
	Medium IBI	47	1.46 (0.87–2.43, *p* = 0.152)	1.31 (0.68–2.51, *p* = 0.422)
	High IBI	60	1.85 (1.11–3.07, *p* = 0.018)	0.65 (0.21–2.00, *p* = 0.451)
NLR	Low NLR	94	–	–
	Medium NLR	48	1.20 (0.73–1.98, *p* = 0.470)	1.37 (0.73–2.57, *p* = 0.320)
	High NLR	35	1.70 (1.01–2.88, *p* = 0.047)	1.57 (0.72–3.46, *p* = 0.258)

Neu, neutrophils; PLT, platelets; CRP, C-reactive protein; NMID, N-terminal mid: fragment of osteocalcin; TPINP, total procollagen type 1 N-terminal propeptide; β-CTX, β-C-terminal telopeptide of type 1 collagen; IBI, inflammatory burden index; NLR, neutrophil to lymphocyte ratio.

### 3.6 Comparative analysis of diagnostic performance of individual and combined tumor bone metabolism markers in detecting bone metastasis

**Figure 8:**
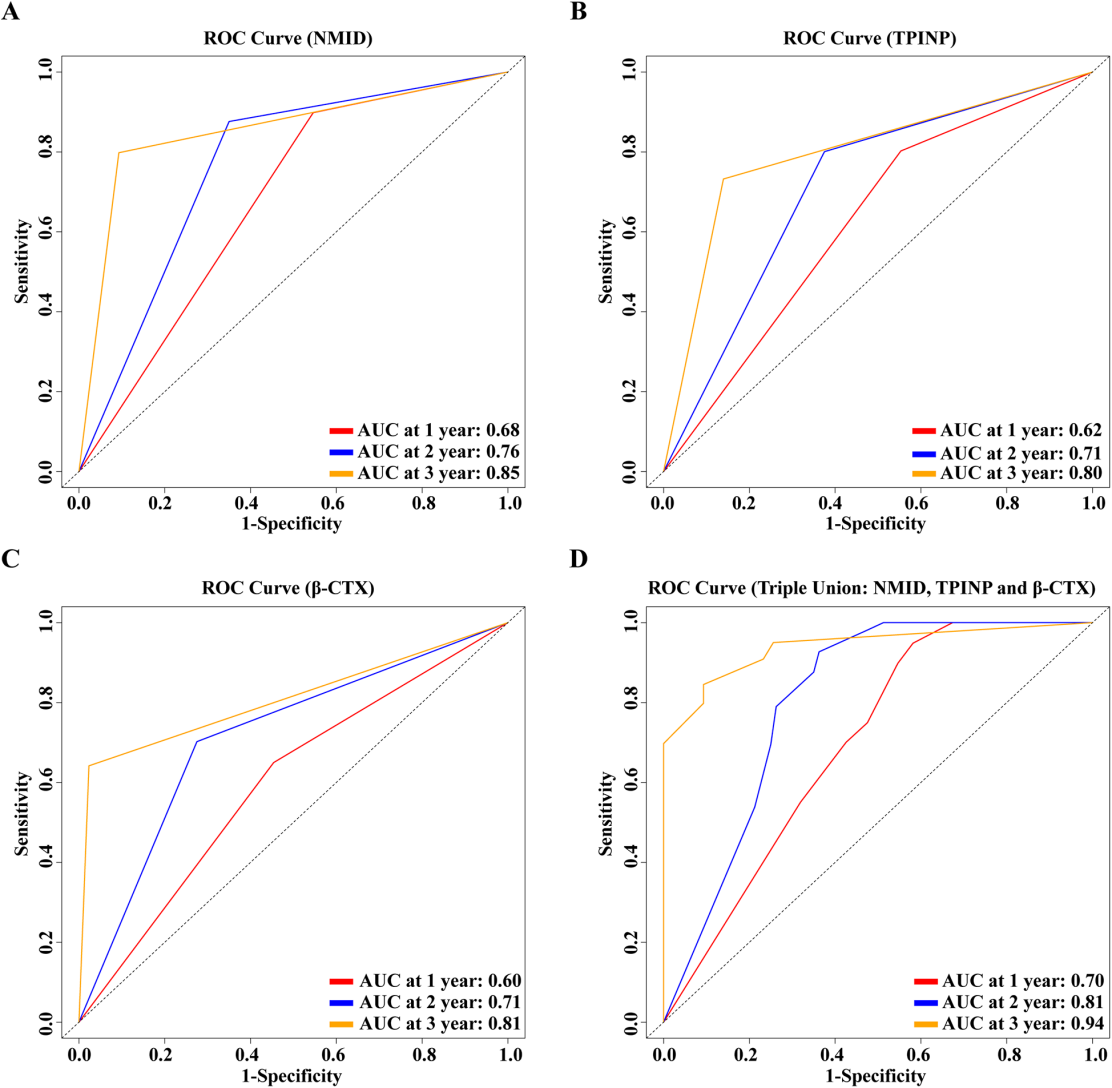
**(A)** 1, 2, 3-year ROC curve for the single-indicator NMID model. **(B)** 1, 2, 3-year ROC curve for the single-indicator TPINP model. **(C)** 1, 2, 3-year ROC curve for the single-indicator β-CTX model. **(D)** 1, 2, 3-year ROC curve for the combined indicators NMID, TPINP, β-CTX model.

In our study, significant differences were observed in the levels of NMID, TPINP, and β-CTX, indicating their potential utility in diagnosing bone metastasis in tumor patients. Specifically, the one-year, two-year, and three-year Area Under the Curve (AUC) values for NMID were 0.68, 0.76, and 0.85, respectively (Figure 8A). For TPINP, these values were 0.62, 0.71, and 0.80, respectively (Figure 8B), over the same periods. β-CTX demonstrated AUC values of 0.60, 0.71, and 0.81 at one, two, and three years, respectively (Figure 8C). Notably, the combined assessment of NMID, TPINP, and β-CTX enhanced the diagnostic accuracy, with the AUC values for the combined markers reaching 0.70, 0.81, and 0.94 at one, two, and three years, respectively (Figure 8D). These findings underscore the superior diagnostic efficacy of the combined use of these biomarkers over individual marker analysis in predicting bone metastasis. The progressive increase in AUC values over time highlights the growing reliability of these markers in long-term patient monitoring, offering a promising avenue for early intervention and tailored treatment strategies in oncological care.

### 3.7 Model validation and assessment

**Figure 9:**
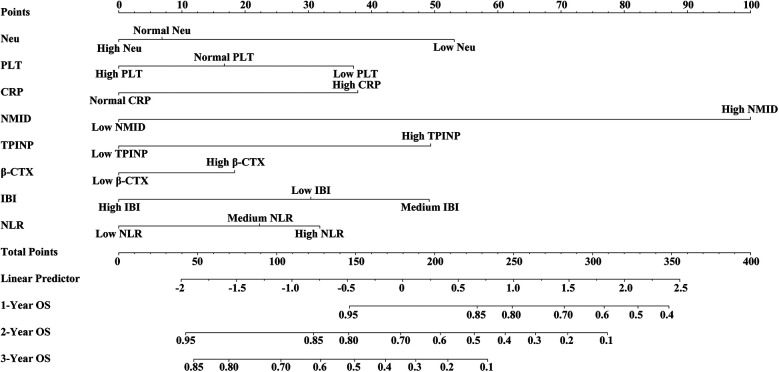
Nomograms reflecting OS based on an in-depth analysis of predictive models.

**Figure 10:**
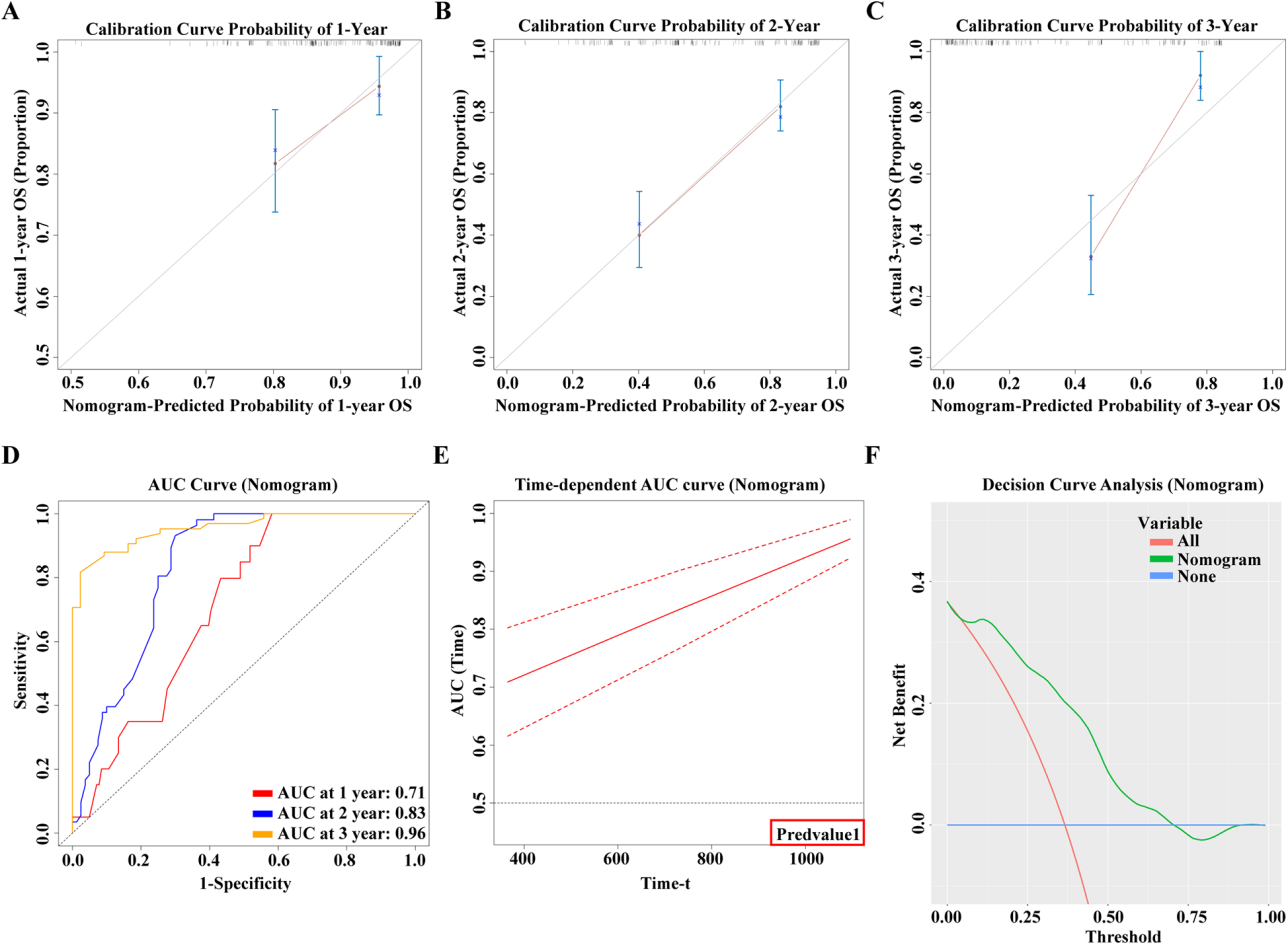
**(A)** 1-year calibration curve of the model. **(B)** 2-year calibration curve of the model. **(C)** 3-year calibration curve of the model. **(D)** 1, 2, 3-year ROC curve of the model. **(E)** Time-AUC curve of the model. **(F)** Clinical Decision Curve Analysis (DCA).

In this study, we meticulously developed nomograms reflecting OS based on an in-depth analysis of predictive models (Figure 9). Utilizing calibration curves, we demonstrated the high congruence between our model's predictions and actual clinical outcomes, underscoring the model's exemplary fit and predictive precision for 1-year, 2-year, and 3-year OS prognoses (Figures 10A–C). This achievement highlights the model's commendable accuracy and reliability in prognostic assessments. Delving further, the Receiver Operating Characteristic (ROC) curves were employed for a nuanced analysis of the OS prognostic prediction model. The nomograms revealed AUC values at various time points as follows: 0.71 for the first year, 0.83 for the second year, and an impressive 0.96 for the third year (Figure 10D). These values not only attest to the model's robustness over diverse temporal milestones but also emphasize its exceptional accuracy in predictive tasks across various timeframes. Moreover, time-dependent ROC curve analysis showcased the nomograms' outstanding performance in predicting OS at distinct temporal intervals. This revelation confirms the model's efficacy in accurately differentiating between positive and negative cases over time, solidifying its exemplary performance in time-dependent prognostic tasks (Figure 10E). To evaluate the clinical applicability of our model, we employed Clinical Decision Curve Analysis (DCA). This analysis assessed the net benefit (NB) derived from implementing the nomograms across a spectrum of threshold probabilities (Figure 10F). The results of this analysis further affirm the model's practical utility and superior predictive prowess within a clinical setting, offering valuable decision-support tools for clinicians in optimizing patient care strategies.

### 3.8 Correlation analysis of bone metabolism biomarkers and cancer pain scores

**Figure 11:**
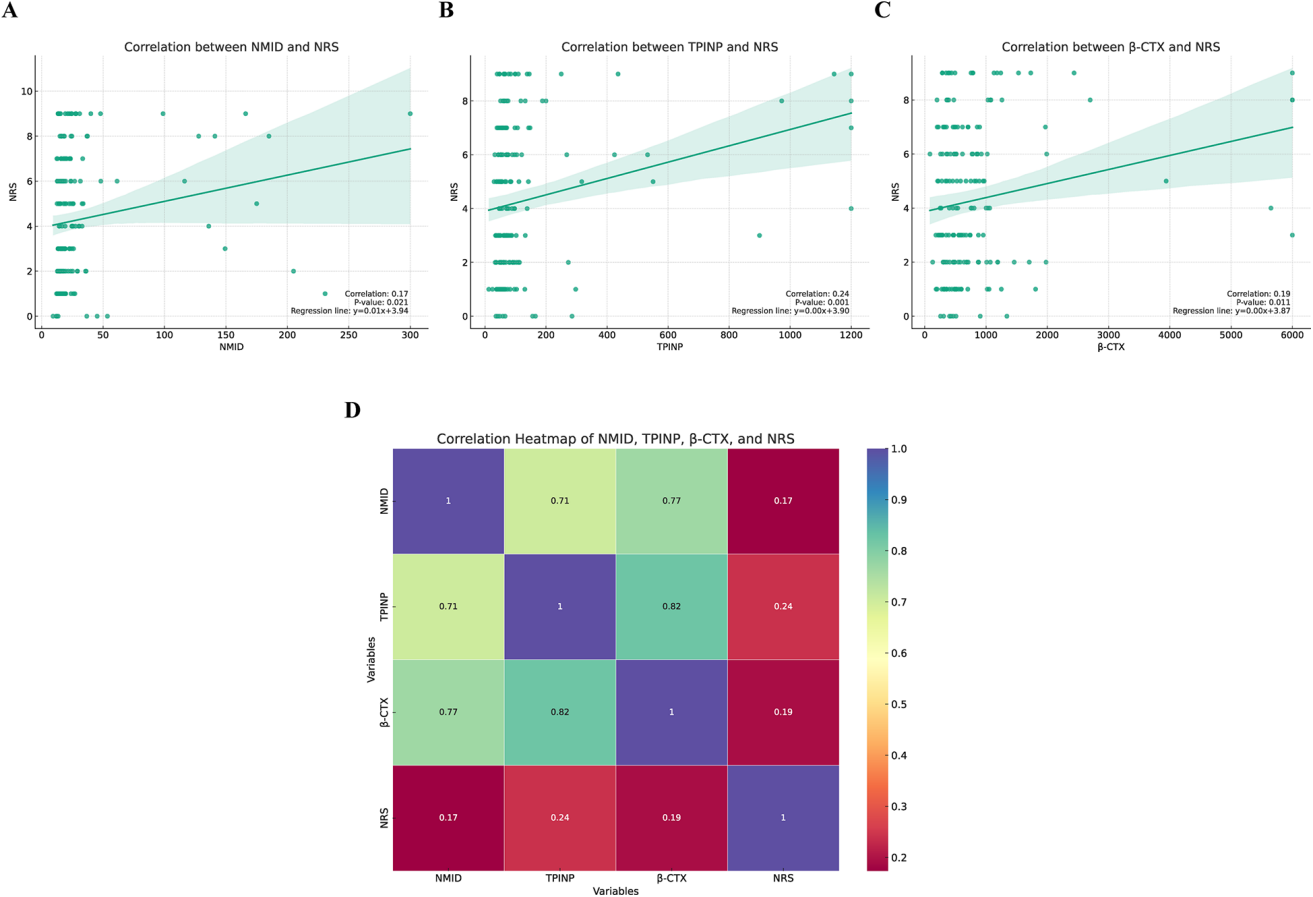
The correlation between three key bone metabolism biomarkers (NMID, TPINP, β-CTX) and numeric rating scale (NRS) pain scores. **(A)** Correlation between NMID and NRS. **(B)** Correlation between TPINP and NRS. **(C)** Correlation between B-CTX and NRS. **(D)** The correlation heatmap depicting the relationships between three key bone metabolism biomarkers (NMID, TPINP, β-CTX) and NRS pain scores.

This study delved into the correlation between three key bone metabolism biomarkers: NMID, TPINP, β-CTX, and cancer pain scores. Employing sophisticated correlation analysis methodologies coupled with heatmap visualization techniques, we meticulously assessed the relationship between the expression levels of these biomarkers and the intensity of cancer pain. The results of the correlation analysis revealed a statistically significant positive correlation between the expression levels of NMID, TPINP, and β-CTX and cancer pain scores. Specifically, the correlation coefficient between NMID expression levels and cancer pain scores was found to be 0.17 (*p* = 0.021), indicating a direct and significant association (Figure 11A). Similarly, TPINP and β-CTX also exhibited significant positive correlations with cancer pain scores, with correlation coefficients of 0.24 (*p* = 0.001) and 0.19 (*p* = 0.011), respectively (Figures 11B,C). Furthermore, the heatmap analysis provided deeper insights into the complex interplay between these biomarkers and their association with cancer pain scores. The heatmap, with its nuanced gradation of colors, visually depicted the degree of correlation between different biomarkers, offering a comprehensive perspective on their role in the progression of cancer (Figure 11D). These findings highlight the critical importance of NMID, TPINP, and β-CTX in the pathophysiology of cancer, particularly in the management of cancer pain. These biomarkers, serving as indicators of disease progression, could potentially guide pain management strategies in cancer patients. Given the impact of cancer pain on patient quality of life, the correlation study of these biomarkers opens new potential avenues for the assessment and treatment of cancer pain.

### 3.9 Development and validation of prognostic models for overall survival and bone metastasis risk in cancer patients

In our groundbreaking study, we have developed two sophisticated prognostic models designed to assess the risk of OS and the likelihood of bone metastasis in cancer patients. These models integrate various clinical biomarkers, offering a quantitative approach for predicting disease progression and patient outcomes with enhanced precision.

$$P=e^{W}/(1+e^{W})$$

*P*: This represents the predicted probability, indicating the likelihood of a certain event occurring.

*e*: This is the base of the natural logarithm, approximately equal to 2.71828.

*W*: Typically, this is a numeric value representing the weighted sum of features calculated by the model.

#### 3.9.1 The OS prognostic risk model is articulated through the following formula

$$\begin{array}{rl} W= & -1.743+0.861\times \mathrm{Normal}\;\mathrm{Neu}+1.502\times \mathrm{High}\;\mathrm{Neu}\\ & -0.119\times \mathrm{Normal}\;\mathrm{PLT}-1.186\times \mathrm{High}\;\mathrm{PLT}\\ & +0.223\times \mathrm{High}\;\mathrm{CRP}+0.933\times \mathrm{High}\;\mathrm{NMID}\\ & +0.282\times \mathrm{High}\;\mathrm{TPINP}+0.182\times \mathrm{High}\;\beta \text{-}\mathrm{CTX}\\ & +0.291\times \mathrm{Medium}\;\mathrm{IBI}-0.483\times \mathrm{High}\;\mathrm{IBI}\\ & +0.444\times \mathrm{Medium}\;\mathrm{NLR}+1.35\times \mathrm{High}\;\mathrm{NLR} \end{array}$$

**Figure 12:**
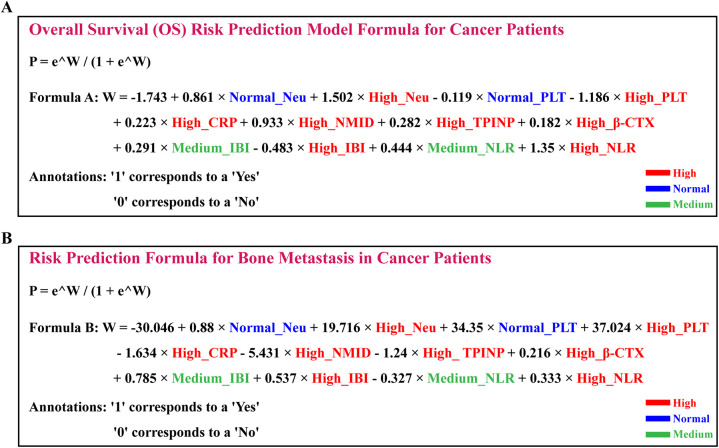
Development and validation of prognostic models for overall survival and bone metastasis risk in cancer patients **(A)** positioned above, the OS prognostic risk model is articulated through the following formula. **(B)** Positioned below, the bone metastasis risk prediction model is encapsulated in the following equation.

This formula is a result of an extensive analysis that combines levels of Neu, PLT, CRP, NMID, TPINP, β-CTX, IBI, and NLR to estimate the OS risk in cancer patients (Figure 12A, Supplementary Table S6).

#### 3.9.2 The bone metastasis risk prediction model is encapsulated in the following equation

$$\begin{array}{rl} W= & -30.046+0.88\times \mathrm{Normal}\;\mathrm{Neu}+19.716\times \mathrm{High}\;\mathrm{Neu}\\ & +34.35\times \mathrm{Normal}\;\mathrm{PLT}+37.024\times \mathrm{High}\;\mathrm{PLT}\\ & -1.634\times \mathrm{High}\;\mathrm{CRP}-5.431\times \mathrm{High}\;\mathrm{NMID}\\ & -1.24\times \mathrm{High}\;\mathrm{TPINP}+0.216\times \mathrm{High}\;\beta \text{-}\mathrm{CTX}\\ & +0.785\times \mathrm{Medium}\;\mathrm{IBI}+0.537\times \mathrm{High}\;\mathrm{IBI}\\ & -0.327\times \mathrm{Medium}\;\mathrm{NLR}+0.333\times \mathrm{High}\;\mathrm{NLR} \end{array}$$

This model strategically amalgamates the expression levels of similar biomarkers to predict the risk of bone metastasis in cancer patients (Figure 12B, Supplementary Table S7).

Both models are underpinned by robust statistical analyses and a wealth of clinical data, aimed at providing a more precise risk assessment to aid clinicians in formulating targeted treatment strategies. These models not only predict the trajectory of the disease but also serve as vital tools for clinical decision-making. This innovative methodology paves the way for personalized treatment and management of cancer patients, promising significant improvements in treatment efficacy and quality of life.

## 4 Discussion

This study has achieved significant breakthroughs in the prediction and diagnosis of bone metastasis in cancer. By establishing a nomogram prediction model based on serum bone metabolism biomarkers, we offer a new perspective and method for the early diagnosis and accurate prognosis of tumor bone metastasis. Additionally, our research revealed a significant correlation between bone metabolism biomarkers and cancer pain scores, providing a novel strategy for understanding and managing the pain in patients with tumor bone metastasis.

Bone metastasis causes tremendous suffering in patients. Previous studies have provided valuable insights into the mechanisms of bone metastasis (5, 11, 25–27). Tumor stem cells undergo a three-step process in the metastasis of lung cancer cells to bone: escape from the primary tumor, entry into the bloodstream, and settlement in the bone (11). Key molecules play a crucial role in this process, directly or indirectly influencing the dynamic microenvironment (26). For example, the nuclear factor-kappa B receptor activator (RANK) axis regulates the activation of osteoclasts during osteolytic lesions (28). Increasing evidence suggests that the bone marrow is one of the most common sites of tumor cell metastasis. The “seed and soil” hypothesis, supported by in-depth studies of bone organics, offers a plausible explanation (29). The settlement of tumor cells (the “seeds”) in the bone marrow (the “soil”) is not a passive process, but actively driven by multiple molecules, activating the signaling pathways of bone metastasis (29). Tumor bone metastasis is a complex cell biological transformation involving various cell types and molecules, including interactions between host cells and the microenvironment, as well as cytokines, adhesion molecules, hormones, and chemokines (30–32). Endothelial cells also play an important role in bone metastasis, stimulating angiogenesis in the extracellular matrix microenvironment to facilitate the migration of tumor cells (33, 34).

Currently, the diagnosis of malignant tumor bone metastasis relies heavily on imaging techniques, each with its limitations. x-rays, though specific, have low sensitivity; SPECT whole-body scans can sensitively, intuitively, and comprehensively reflect changes in bone metabolism but lack specificity; PET-CT, while accurate, is expensive; and CT and MRI are also costly and limited in scanning range. Bone metabolism biomarkers reflect the metabolic state of bones. During tumor bone metastasis, the bone remodeling process accelerates, leading to an increased rate of bone metabolism (11). This change often precedes the morphological changes detected by imaging studies. The bone metabolism process, including bone resorption and formation, and related biomarkers can predict tumor deterioration (11). In osteolytic lesions, the balance between osteoblast-led bone formation and osteoclast-dominated bone resorption is disrupted, leading to the production of related bone metabolism biomarkers (35). Osteocalcin (OC) is a small, globular protein specifically synthesized and secreted by non-proliferating osteoblasts (36). Under the influence of calcium ions, the carboxylated glutamic acid of OC promotes its binding with hydroxyapatite and deposition in the extracellular bone matrix, thus facilitating bone development (36). Studies show that OC has the capability to attract and activate osteoclasts; its carboxylated end chemically induces osteoclast precursors, thereby regulating bone resorption (37, 38). A portion of OC is released into the bloodstream, and about one-third of OC in serum is intact, another third is short peptides of amino acids, and the remaining third consists of cleaved NMID fragments. NMID in serum is stable and reflects the level of osteocalcin in the bone, thereby indicating the activity of osteoblasts and the status of bone formation and resorption (39). It is a bone metabolism marker with high sensitivity and specificity. β-CTX is a terminal peptide generated during the degradation of type I collagen fibers by osteoclasts during bone resorption, indicative of bone matrix absorption and osteoclast activity (40, 41). The newly synthesized carboxy-terminal peptide of type I collagen is alpha-type (*α*-CTX), while the mature form is predominantly β-CTX (40, 41). Released into the bloodstream, β-CTX is excreted directly through urine without undergoing renal or hepatic metabolism, making it a sensitive and specific marker (41). TPINP reflects osteoblast activity and bone formation rate and is recognized as a bone formation marker. Type I collagen, a crucial component of bone matrix, is broken down into TPINP fragments during the maturation of type I procollagen into type I collagen (42, 43). TPINP reflects the synthesis of type I collagen and serves as a sensitive and specific indicator of bone formation (42, 43). Monitoring these bone metabolism biomarkers allows for a deeper understanding of the process of tumor bone metastasis, providing crucial information for early diagnosis and treatment.

Our study indicates that NMID, TPINP, and β-CTX, as individual markers, hold significant predictive value for tumor bone metastasis. Their expression levels in patients with tumor bone metastasis are significantly higher than those in the non-metastatic group and healthy controls, further affirming their critical role in tumor bone metastasis. Particularly, when these three markers are used in combination, the predictive model's AUC value significantly increases, suggesting that these biomarkers, when used together, possess higher sensitivity and specificity in predicting the risk of tumor bone metastasis. The changes in bone metabolism biomarkers not only reveal the occurrence of bone metastasis but also reflect the interaction between tumor cells and the host microenvironment. The settlement and growth of tumor cells in the bone marrow microenvironment depend not only on their own biological characteristics but are also influenced by host environmental factors. The variations in biomarkers such as NMID, TPINP, and β-CTX indicate a disrupted dynamic balance between bone resorption and formation in the process of bone metastasis. The elevation of these biomarkers suggests intensified bone destruction and remodeling, providing new insights into the molecular mechanisms of tumor bone metastasis.

The Nomogram model developed in this study not only enhances the diagnostic accuracy of tumor bone metastasis but also provides robust support for clinical decision-making. By accurately assessing a patient's risk of bone metastasis, physicians can more rationally formulate treatment plans and monitoring strategies. Early intervention and treatment are especially crucial for high-risk patients, contributing to improved quality of life and prognosis. Moreover, our research has uncovered a significant correlation between bone metabolism biomarkers and cancer pain scores, offering a new perspective in understanding the pain experienced by patients with tumor bone metastasis. The positive correlation between the levels of biomarkers like NMID, TPINP, and β-CTX, and cancer pain scores suggests that these markers not only reflect bone metabolism status but may also be directly linked to tumor-induced bone pain. This finding is vital for improving pain management and enhancing the quality of life in cancer patients. By monitoring these biomarkers, physicians could more accurately assess the pain levels in tumor patients and accordingly adjust treatment protocols, such as optimizing pain control and bone-protective therapies.

Despite the positive outcomes of this study, the mechanisms of tumor bone metastasis remain complex and variable, necessitating further in-depth research. Future work should focus on comprehensively understanding the interactions between tumor cells and the bone marrow microenvironment and exploring additional potential bone metabolism biomarkers. Furthermore, in-depth studies on patients with different types of tumors and clinical stages are required to validate and optimize the predictive model's applicability and accuracy. It is important to acknowledge that since this is a retrospective study, biases influencing the causality between biomarkers and outcomes may be present. The retrospective design introduces potential selection bias, information bias, and confounding factors, which could affect the interpretation of the observed associations between biomarkers and tumor bone metastasis. These biases may limit the ability to definitively establish causal relationships, and future prospective studies will be essential to more rigorously assess the temporal dynamics and causality between biomarkers and bone metastasis. Our research provides new insights into the pain mechanisms of tumor bone metastasis, including the acidic microenvironment caused by tumors and osteoclasts, cytokines released by the tumor and stroma, and neuropathic factors. A deeper understanding of these complex mechanisms will aid in developing more effective treatment strategies to alleviate pain and improve the quality of life for patients. However, overcoming the limitations of the retrospective design and addressing the aforementioned biases in future studies will be crucial in refining the predictive models and validating these pain mechanisms.

## 5 Conclusion

In conclusion, this study contributes significantly to the prognosis assessment and pain management of cancer patients by constructing a risk prediction model and revealing the correlation between bone metabolism biomarkers and cancer pain scores. These findings not only enhance the accuracy of diagnosis and prognosis assessment of tumor bone metastasis but also provide new directions for clinical treatment. Our research underscores the importance of adopting multifaceted and comprehensive strategies in cancer therapy and the necessity of exploring molecular mechanisms and potential applications of biomarkers in future oncological research.

## Data Availability

The original contributions presented in the study are included in the article/Supplementary Material, further inquiries can be directed to the corresponding author.

## Supplementary material

Supplementary Table S1 Baseline clinical-pathological and laboratory examination results of patients.

Supplementary Table S2 Chi-square test analysis demonstrating significant associations between the occurrence of bone metastasis in tumors and various clinical-pathological features as well as laboratory examinations.

Supplementary Table S3 Chi-square test analysis demonstrating significant associations between the levels of NMID and various clinical-pathological features as well as laboratory examinations.

Supplementary Table S4 Chi-square test analysis demonstrating significant associations between the levels of TPINP and various clinical-pathological features as well as laboratory examinations.

Supplementary Table S5 Chi-square test analysis demonstrating significant associations between the levels of β-CTX and various clinical-pathological features as well as laboratory examinations.

Supplementary Table S6 The OS prognostic risk model is formulated using a linear regression statistical approach, as described by the following formula.

Supplementary Table S7 The bone metastasis risk prediction model, incorporating linear regression statistical methods, is encapsulated in the following equation.
